# Keyhole retrosigmoid approach for microvascular decompression surgery: Systematic review and single-arm meta-analysis

**DOI:** 10.1007/s10143-026-04178-0

**Published:** 2026-02-17

**Authors:** Valentin F. Weiger, Florian S. Halbeisen, Felipe Constanzo, Michel Roethlisberger, Jehuda Soleman, Luigi Mariani, Raphael Guzman, Jonathan Rychen

**Affiliations:** 1 Department of Neurosurgery, University Hospital of Basel, Spitalstrasse 21, Basel, 4031 Switzerland; 2 Surgical Outcome Research Center Basel, University Hospital of Basel, Basel, Switzerland; 3 Department of Skull Base Surgery, Clinica Bio Bio, Concepcion, Chile

**Keywords:** Keyhole, Burr hole, Retrosigmoid, Microvascular decompression, Trigeminal neuralgia, Hemifacial spasm

## Abstract

Microvascular decompression (MVD) surgery is traditionally performed via a retrosigmoid craniotomy. Recently, neurosurgeons have increasingly adopted smaller approaches; however, the available evidence has not yet been systematically compiled. This is the first systematic review and meta-analysis on the efficacy and safety of ≤ 2-cm-keyhole retrosigmoid MVD. PubMed and Embase databases were searched for studies involving adults with trigeminal neuralgia (TN), hemifacial spasm (HFS), or glossopharyngeal neuralgia (GPN), who underwent first-time retrosigmoid keyhole MVD (diameter ≤ 2 cm). Primary outcomes were symptom relief and complications. Study quality was assessed using the Methodological Index for Non-Randomized Studies (MINORS). Pooled estimates were calculated using a random-effects single-arm meta-analysis. 32 publications met the inclusion criteria. 93.7% had a retrospective design. The mean MINORS score was 9.9 ± 1.4 out of 16. Among 5,883 patients, 37.7% had TN, 61.6% HFS, and 0.7% GPN. The complete symptom resolution rate was 89.8% (95% CI [0.855–0.930]) for TN, 91.1% (95% CI [0.871–0.940]) for HFS and 88.7% (95% CI [0.746–0.954]) for GPN. The overall complication rate was 13.9% (95% CI, 0.107–0.180), with a permanent neurological deficit rate of 4.7% (95% CI, 0.030–0.074), all involving cranial nerves (CN). In patients with TN, the most frequent permanent neurological deficit was a sensory CN V deficit, occurring in 2.6% (95% CI, 0.013–0.050), followed by a CN VIII deficit in 1.9% (95% CI, 0.011–0.035). Among HFS patients, the most frequent permanent deficits were CN VIII in 5.0% (95% CI, 0.026–0.094) and CN VII in 2.1% (95% CI, 0.008–0.050). None of the studies described cases requiring conversion from a keyhole approach to a standard craniotomy. The keyhole retrosigmoid approach for MVD appears to be safe and effective. Comparative studies are needed to evaluate potential advantages of a keyhole approach over a standard craniotomy.

**Supplementary Information:**

The online version contains supplementary material available at https://doi.org/10.1007/s10143-026-04178-0.

## Introduction

Neurovascular compression syndromes (NVCS) of the cerebellopontine angle are characterized by paroxysmal craniofacial pain or spasm. The most common syndromes are trigeminal neuralgia (TN) and hemifacial spasm (HFS), followed by glossopharyngeal neuralgia (GPN) [19, 39]. In patients with an identified neurovascular conflict, microvascular decompression (MVD) surgery is the treatment of choice when pharmacological therapy fails or botulinum toxin injections are not effective enough [2]. MVD surgery consists in the separation of the offending vessel from the cranial nerve (CN) to relieve neurovascular compression [16]. First suggested in the early 1950 s, it was refined and popularized in the second half of the 20th century, most notably by Peter Jannetta [25, 27, 51]. MVD surgery as well as internal neurolysis (IN) and partial rhizotomy (PR) are traditionally performed via a retrosigmoid craniotomy [6]. In the early years, the bone window measured approximately 5 centimeters (cm) in diameter [25, 33]. However, the size of the bone window has gradually decreased since the late 1990 s [23, 44]. With recent advances in visualization techniques and the development of tube shaft micro-instruments, neurosurgeons have adopted increasingly smaller approaches based on the keyhole concept, reducing the bone window diameter to 2 cm or less [7, 18, 40, 41]. A substantial body of literature on MVD for NVCS reports the use of the keyhole retrosigmoid approach [21, 30]. However, to the best of our knowledge, no effort has yet been made to compile all the existing data on this topic. This study presents the first systematic review and meta-analysis on the efficacy and safety of MVD performed through a ≤ 2-cm keyhole retrosigmoid approach.

## Materials and methods

### Literature research

This systematic review and single-arm meta-analysis was conducted in accordance with the Preferred Reporting Items for Systematic Reviews and Meta-Analyses (PRISMA) 2020 Guidelines and followed the population, intervention, control and outcome (PICO) scheme [37, 47]. The analyzed population consisted of patients with TN, HFS or GPN. The interventions considered were MVD surgery, IN and PR through a keyhole retrosigmoid approach of ≤2 cm. Primary outcomes were the efficacy (i.e. symptom relief) and safety (i.e. complication rates) of this approach. PubMed and Embase databases were searched on the 21st of March 2025. The literature search was structured into two main search queries: the first one was a combination of the MeSH term “MVD surgery” and the three NVCS given above, whereas the second one was a combination of “retrosigmoid”, “keyhole”, “burr hole” and “minimally invasive” (see Supplementary Data). The search was adapted to Embase notation with the aid of a query translator [12, 13, 42]. Additionally, reference lists of key primary studies and relevant systematic reviews were manually screened for additional articles.

### Eligibility criteria and study selection process

All peer-reviewed English full text articles published until March 2025 were screened for inclusion. Studies were eligible if they investigated patients over 18 years of age suffering from either TN, HFS, or GPN. Only cases involving first-time MVD surgery, with or without IN or PR, through a retrosigmoid keyhole approach with a bone window diameter of ≤2 cm were considered. We chose a cut-off of 2 cm because most studies reporting outcomes of the keyhole retrosigmoid approach used a bone opening diameter less than or equal to 2 cm. Eligible studies were required to report on both efficacy and safety, with a minimum follow-up (FU) duration of one month. Acceptable study designs encompassed observational studies, case series, comparative cohort studies, and technical notes. Studies were excluded if patients had atypical clinical presentations (e.g., multiple or bilateral NVCS) or secondary causes of their condition (e.g., multiple sclerosis or tumor). Studies were excluded if the bone window diameter was not reported or if the size was given only as an area. Additionally, patients who had undergone prior procedures – such as percutaneous interventions, radiosurgery, or peripheral neurectomy – were excluded. Case reports, conference abstracts, reviews, and meta-analyses were not considered for analysis. All abstracts were screened by the first author (VFW). Any abstracts with uncertain eligibility were independently reviewed by the last author (JR). Duplicate removal was performed with Zotero Reference Manager (Roy Rosenzweig Center for History and New Media, https://www.zotero.org) and manually double-checked by the first author.

### Assessment of quality and risk of bias

The methodological quality of the included studies was assessed using the Methodological Index for Non-Randomized Studies tool (MINORS), a tool specifically developed for surgical research [49]. We applied the 8-item version of the MINORS tool to all included studies, rather than the 12-item comparative version, as our review did not rely on comparative effect estimates to draw its conclusions. Quality of the included studies was classified as good (MINORS 15–16), moderate (9–14), or poor (≤ 8). Additionally, each study was independently assessed for selection, confounding, performance, detection, attrition, and reporting biases (see Supplementary Table 1).

### Data extraction

For each eligible study, the following study characteristics were extracted: study design, country of origin and year of publication. Additionally, data regarding the number of patients and specific type of treated NVCS were collected. Demographic and preoperative variables included sex, age and duration of symptoms. Operative details such as bone window diameter, type of procedure, visualization technique, offending vessels and operating time were documented. Postoperative data included, amongst others, duration of FU, treatment efficacy, surgical complications, and need for re-operation.

### Quantitative synthesis and statistical analysis

Descriptive statistics were used to summarize the baseline and surgical characteristics of the included studies. For continuous variables, weighted means and ranges were calculated, while categorical variables were reported as absolute counts and percentages. We assessed the main outcomes, i.e. efficacy and safety, at the last reported FU. Efficacy for each NVCS was categorized into three groups: complete resolution of symptoms, partial resolution defined as improvement of symptoms without complete resolution, and failure defined as no change or worsening of symptoms. Additionally, recurrences were documented and included in the efficacy synthesis. All procedure-related complications were extracted and categorized, and neurological deficits were classified as transient or permanent. In cases where permanence was not specified, neurological deficits were conservatively classified as permanent. When IN was performed instead of MVD, or when MVD was combined with PR, postoperative deficits of the affected CN were not considered complications, as these are expected outcomes of these interventions.

To calculate pooled estimates of efficacy, recurrence, and complication rates, we conducted a random-effects meta-analysis of proportions with the *meta* package in R (R Core Team, https://www.r-project.org). A random-effects model was chosen to account for inter-study variation, thereby providing estimates that better reflect outcomes across diverse populations. We applied a logit transformation with inverse-variance weighting and introduced a continuity correction when event rates were 0 or 1. Pooled estimates with *p* < 0.05 were considered statistically significant, corresponding to 95% confidence intervals. Between-study heterogeneity was quantified with the I² statistic and τ², and its statistical significance was tested with Cochran’s Q test, with *p* < 0.1 as the threshold for significance. Subgroup differences between NVCS subtypes were assessed using the Q test for subgroup differences, and forest plots were generated to illustrate individual study results together with subgroup summary effects.

## Results

### Study identification and selection

**Fig. 1:**
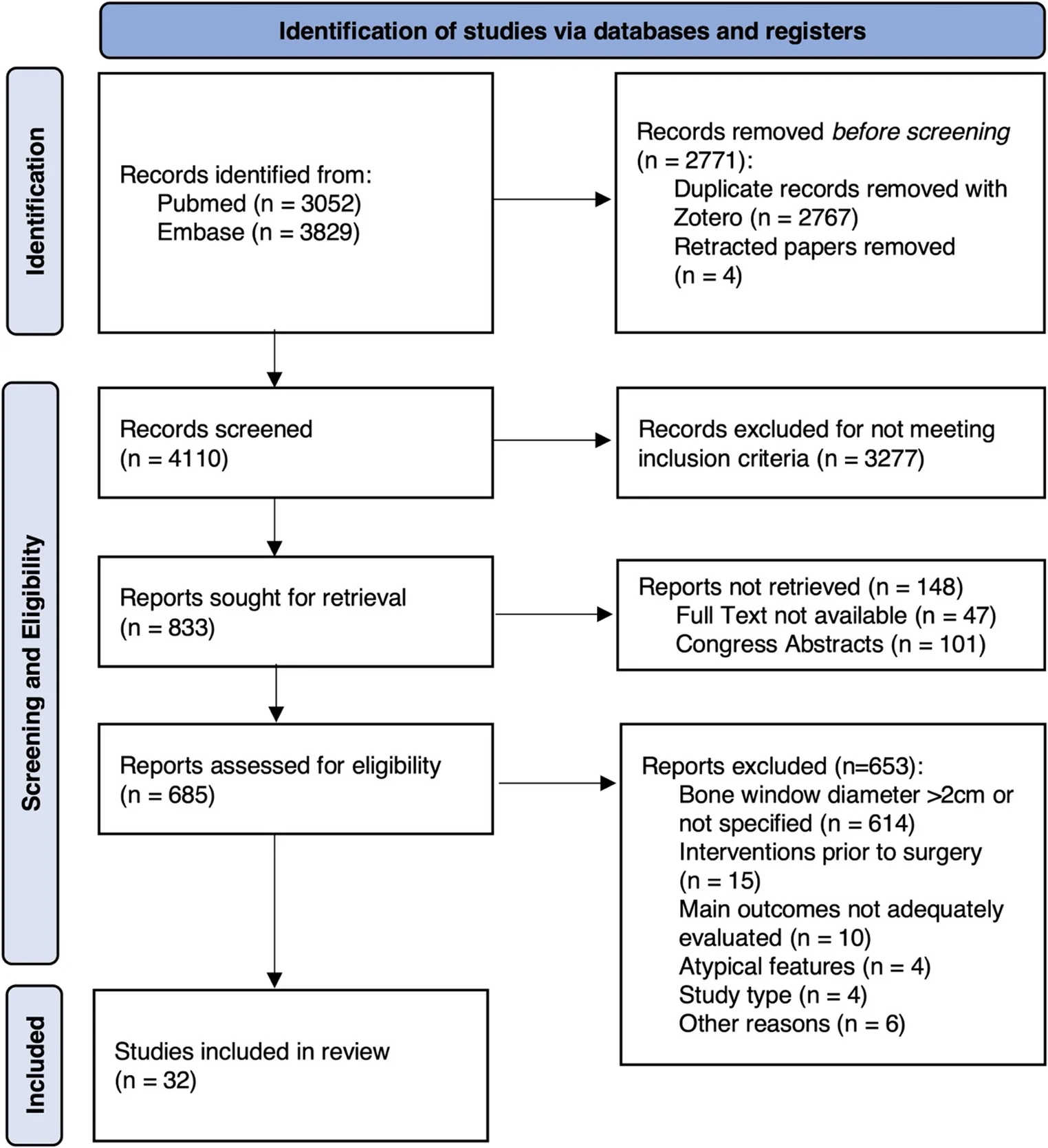
Preferred Reporting Items for Systematic Reviews and Meta-Analysis (PRISMA) diagram illustrating the individual steps of literature research and the study selection process. Generated using Microsoft Word (Microsoft Corporation, Redmond, WA, USA)

The full study identification and selection process is summarized in Fig. 1 [37]. A total of 32 full-text articles met the inclusion criteria and were included in the meta-analysis [1, 5, 8–11, 14, 15, 17, 18, 21, 24, 26, 28–31, 35, 38, 45, 46, 50, 52, 54–62, 64]. 

### Qualitative synthesis

Of the included publications, 23 (71.9%) were observational studies, and 9 (28.1%) were comparative cohort studies. Thirty studies (93.7%) had a retrospective design, while 2 (6.3%) were prospective. The mean FU time was 3.9 years (range: 0.1–12.0). The mean MINORS score was 9.9 ± 1.4 out of 16 (range: 7–12), indicating an overall moderate quality of the included studies. The risk-of-bias assessment indicated a generally high risk of selection, confounding, performance, and detection biases, but a relatively low risk of attrition and reporting biases. The detailed qualitative assessments are provided in Supplementary Table 1.

### Quantitative synthesis

#### Baseline and surgical characteristics 

The 32 included studies encompassed a total of 5883 patients: 2219 with TN (37.7%), 3625 with HFS (61.6%), and 39 with GPN (0.7%). MVD accounted for the vast majority of procedures (*n* = 5825; 99.0%), whereas IN and PR were only used in a small minority of cases. Microscopic visualization was employed in 72.5% of cases (*n* = 4263), while fully endoscopic and endoscopic-assisted techniques were used in 12.8% (*n* = 754) and 14.6% (*n* = 859) of cases, respectively. Among 31 studies that reported the interposition material (*n* = 5585), all used a synthetic material (e.g., Teflon^®^). More detailed baseline and surgical characteristics of the pooled cohort are provided in Table 1.

**Table 1:** Baseline information and surgical characteristics. Patient demographics, NVCS characteristics, and surgical details of the pooled cohort are presented. Additionally, for each variable, the number of studies in which corresponding data were available is reported

Variable	Value *n* (%)			Available studies (number of patients ≙ %)
NVCS type				32 (5883 ≙ 100%)
TN	2219 (37.7%)			15
HFS	3625 (61.6%)			17
GPN	39 (0.7%)			2
Sex				28 (5224 ≙ 88.8%)
Female	3157 (60.4%)			
Male	2067 (39.6%)			
Weighted mean age (range) [years]	55.3 (19–89)			24 (4658 ≙ 79.2%)
Weighted mean symptom duration (range) [years]	5.4 (0.2–35)			18 (4303 ≙ 73.1%)
Weighted mean FU time (range) [years]	3.9 (0.1–12)			17 (2764 ≙ 47.0%)
Loss to FU	116 (2.0%)			32 (5883 ≙ 100%)
Procedure type				32 (5883 ≙ 100%)
MVD	5825 (99.0%)			
IN	43 (0.7%)			
MVD + PR	15 (0.3%)			
Visualization technique				32 (5883 ≙ 100%)
Microscopic	4263 (72.5%)			
Endoscopic-assisted	859 (14.6%)			
Fully endoscopic	754 (12.8%)			
Exoscopic	7 (0.1%)			
Offending vessel	TN	HFS	GPN	26 (3900 ≙ 66.3%)
SCA^a^	414 (33.0%)	–	–	
AICA^b^	65 (5.2%)	939 (36.1%)	5 (12.8%)	
PICA^c^	2 (0.2%)	673 (25.8%)	27 (69.2%)	
VA or BA^d^	28 (2.2%)	58 (2.2%)	3 (7.7%)	
Vein	108 (8.6%)	3 (0.1%)	–	
Multiple vessels	261 (20.8%)	845 (32.4%)	4 (10.3%)	
Other	378 (30.1%)	87 (3.3%)	–	

^a^ SCA: Superior cerebellar artery

^b^ AICA: Anterior inferior cerebellar artery

^c^ PICA: Posterior inferior cerebellar artery

^d^ VA or BA: Vertebral or basilar artery

#### Primary outcome: efficacy

**Fig. 2:**
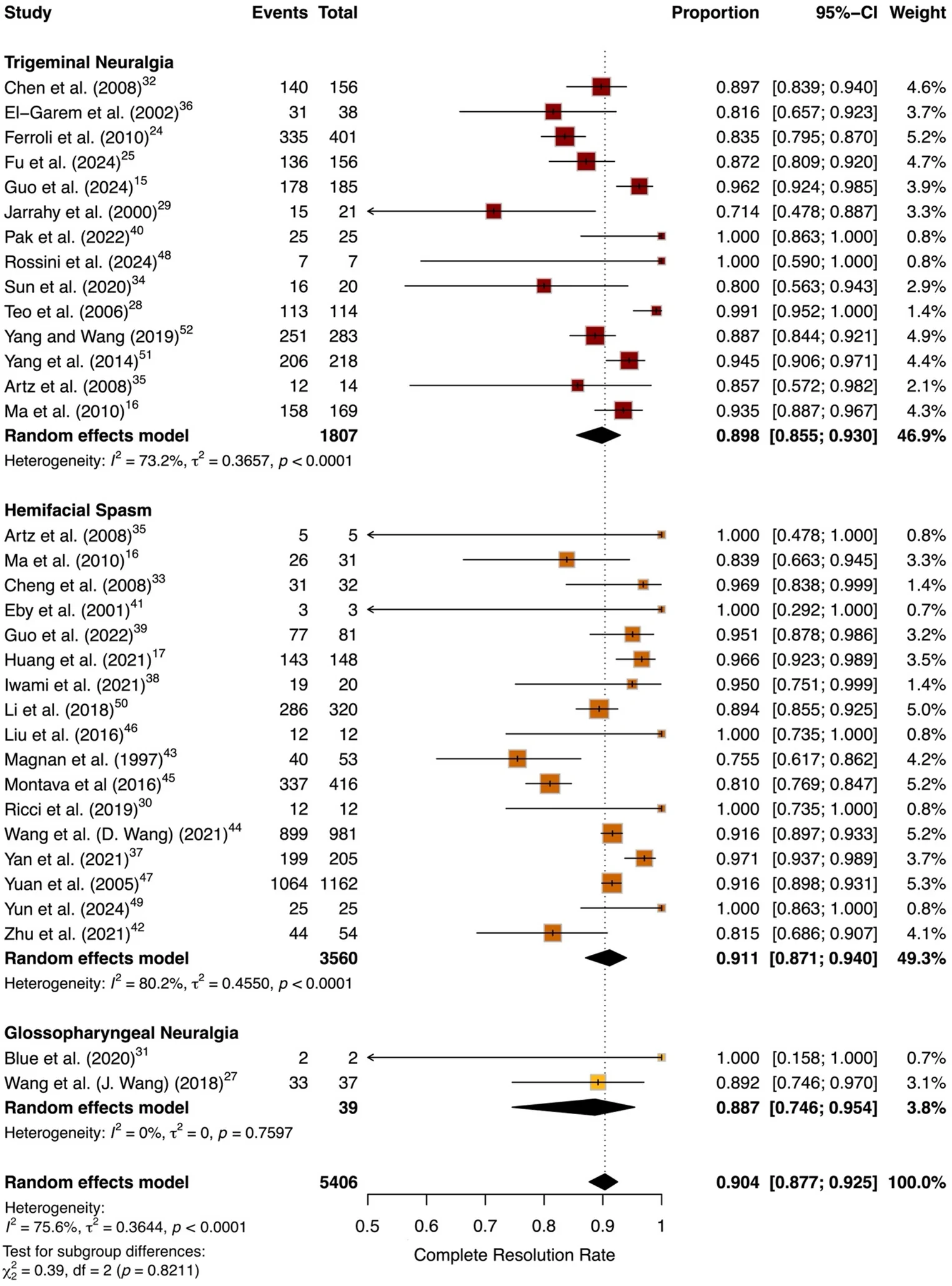
Forest plot of complete resolution rates. Each included study is shown with the first author’s name, the number of patients with complete resolution of symptoms (events), and total number of patients enrolled. The plot depicts the point estimate of the complete resolution rate for each study, together with its 95% CI and study weight in the meta-analysis. Studies are grouped according to NVCS types. Overall pooled results of the random-effects meta-analysis, along with heterogeneity and potential subgroup differences are presented. Generated using R (R Core Team, https://www.r-project.org)

**Fig. 3:**
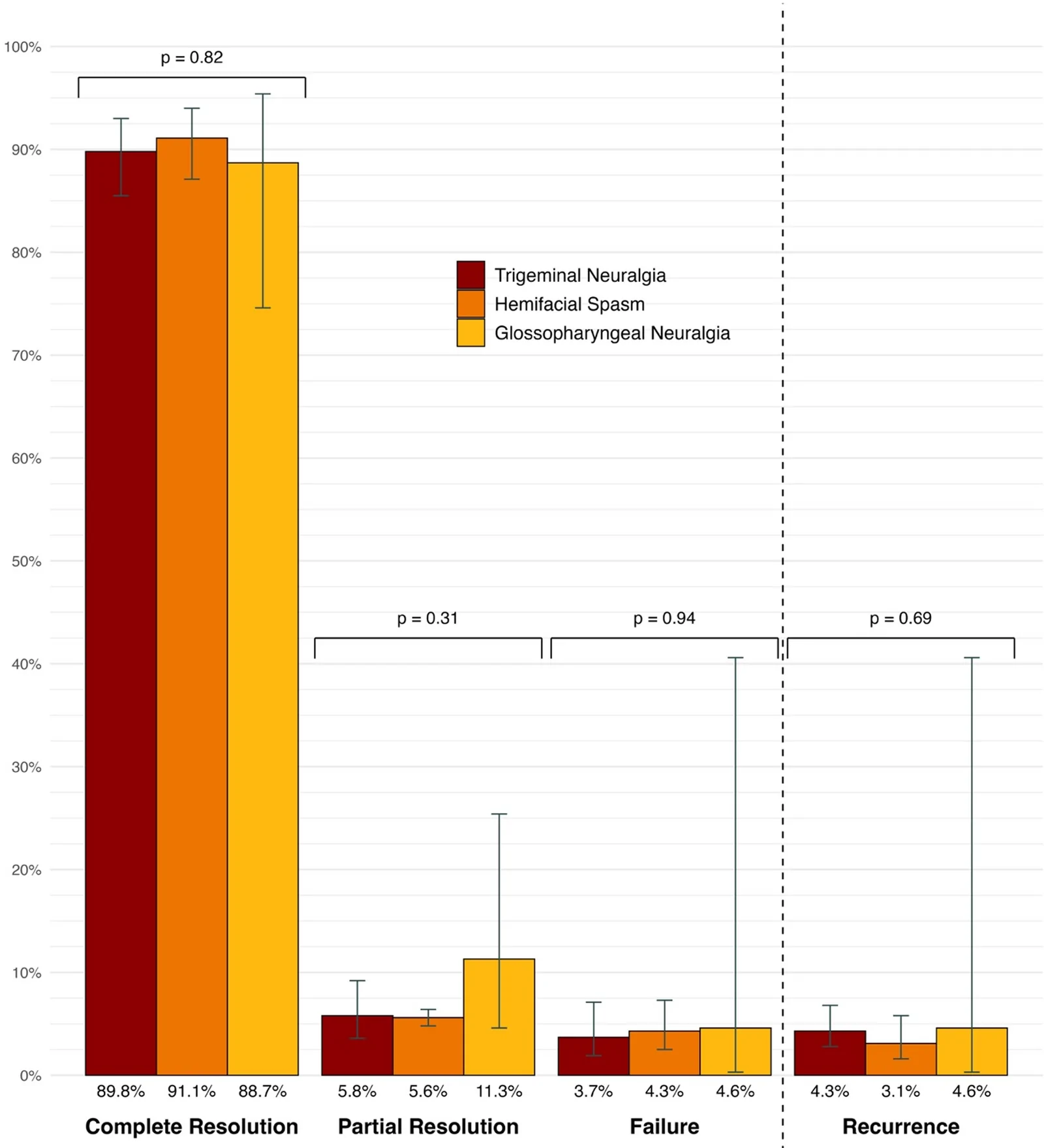
Grouped bar chart illustrating efficacy outcome. Each bar cluster corresponds to an efficacy category (complete resolution, partial resolution, failure, and recurrence). Within each cluster, separate bars represent the point estimate of the pooled rate for each NVCS, with whiskers indicating the 95% CI. The p-values shown for each cluster correspond to the test for subgroup differences analogous to the forest plots. While the complete resolution, partial resolution, and failure rates sum to 100% within each individual study, the pooled rates from the meta-analysis may not add up to 100%, since they are calculated independently for each outcome (random-effects model). Generated using R

Based on a random-effects meta-analysis, the complete resolution rate across all available studies (31, *n* = 5406) was 90.4% (95% CI [0.855–0.930]). NVCS-specific rates were 89.8% (95% CI [0.855–0.930]) for TN, 91.1% (95% CI [0.871–0.940]) for HFS and 88.7% (95% CI [0.746–0.954]) for GPN. Forest plots of the NVCS-specific complete resolution rates are shown in Fig. 2. The overall partial resolution rate was 5.6% (95% CI [0.045–0.069]) across 27 studies (*n* = 4849), whilst the failure rate was 4.1% (95% CI [0.028–0.061]) across 26 studies (*n* = 4448). The overall recurrence rate was estimated at 3.9% (95% CI [0.028–0.054]) based on 30 available studies (*n* = 5069). The different efficacy rate categories for each NVCS are presented in Fig. 3. Neither the efficacy rates nor the recurrence rates differed significantly among the various NVCS. The reoperation rate was inconsistently reported (with 14 studies lacking this information), and therefore not analyzed.

#### Primary outcome: safety

**Fig. 4:**
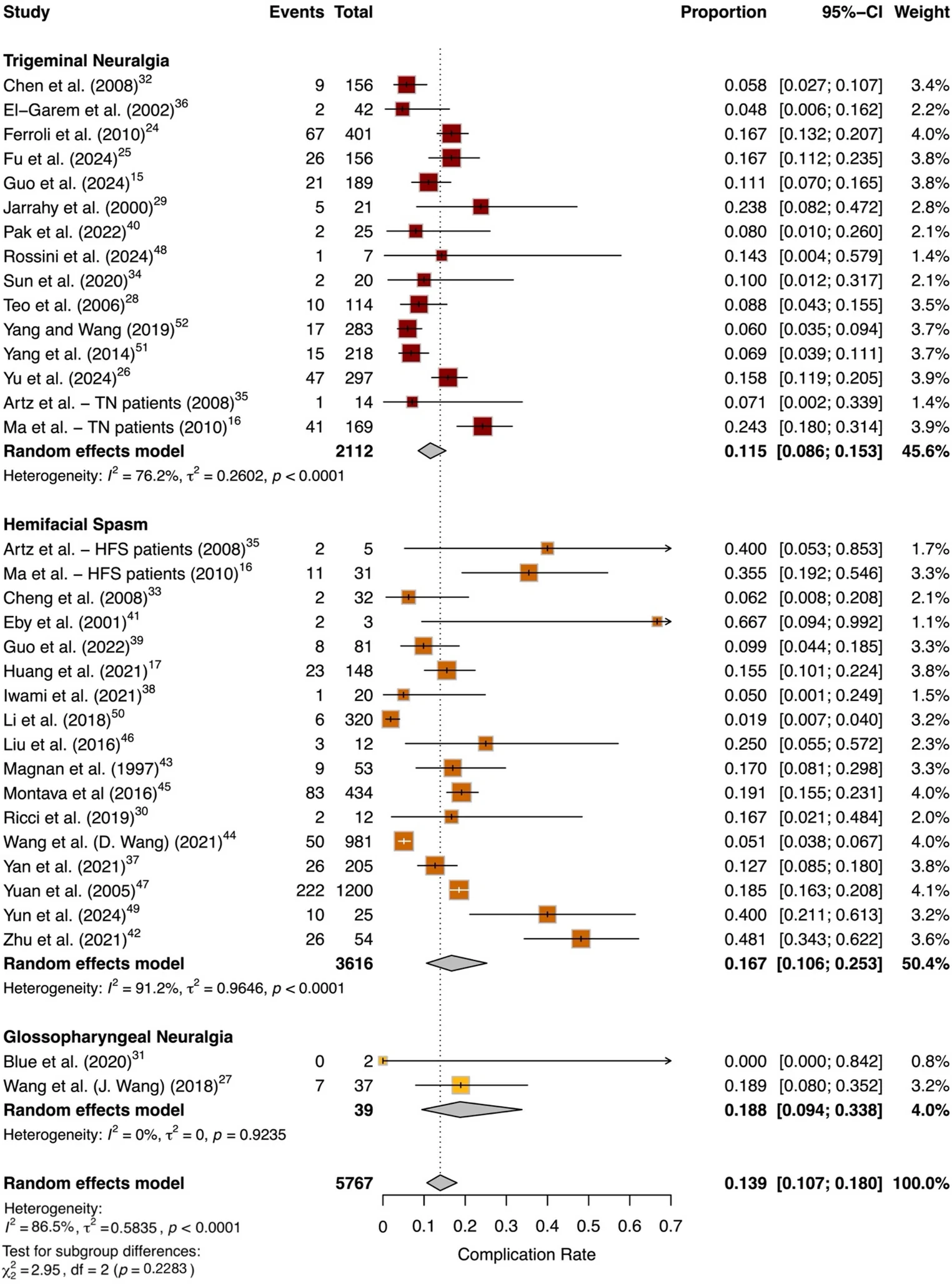
Forest plot of complication rates. Each included study is shown with the first author’s name, the number of patients with any surgical complication (events), and total number of patients enrolled. The plot depicts the point estimate of the complication rate for each study, together with its 95% CI and study weight in the meta-analysis. Studies are grouped according to NVCS types. Overall pooled results of the random-effects meta-analysis, along with heterogeneity and potential subgroup differences are presented. Generated using R

**Fig. 5:**
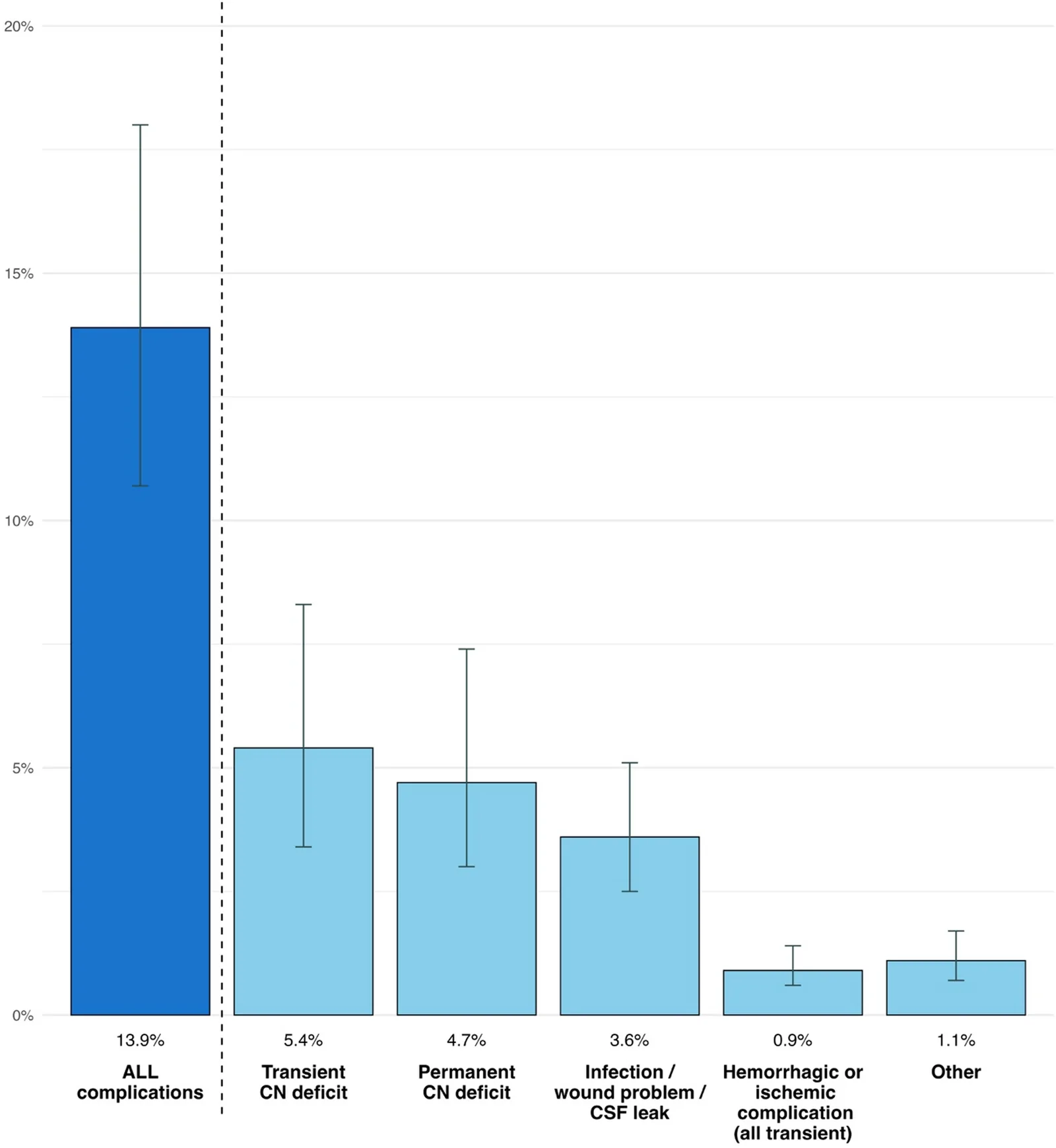
Bar chart of overall and category-specific complication rates. Each bar represents the point estimate of the pooled rate for the respective complication category, with whiskers indicating the 95% CI. The bar to the left of the dashed vertical line represents the overall complication rate, whereas the bars to the right show the rates for specific complication categories. The “Other” category includes complications such as ataxia, dislodged Teflon pieces or tension pneumocephalus. Generated using R

**Fig. 6:**
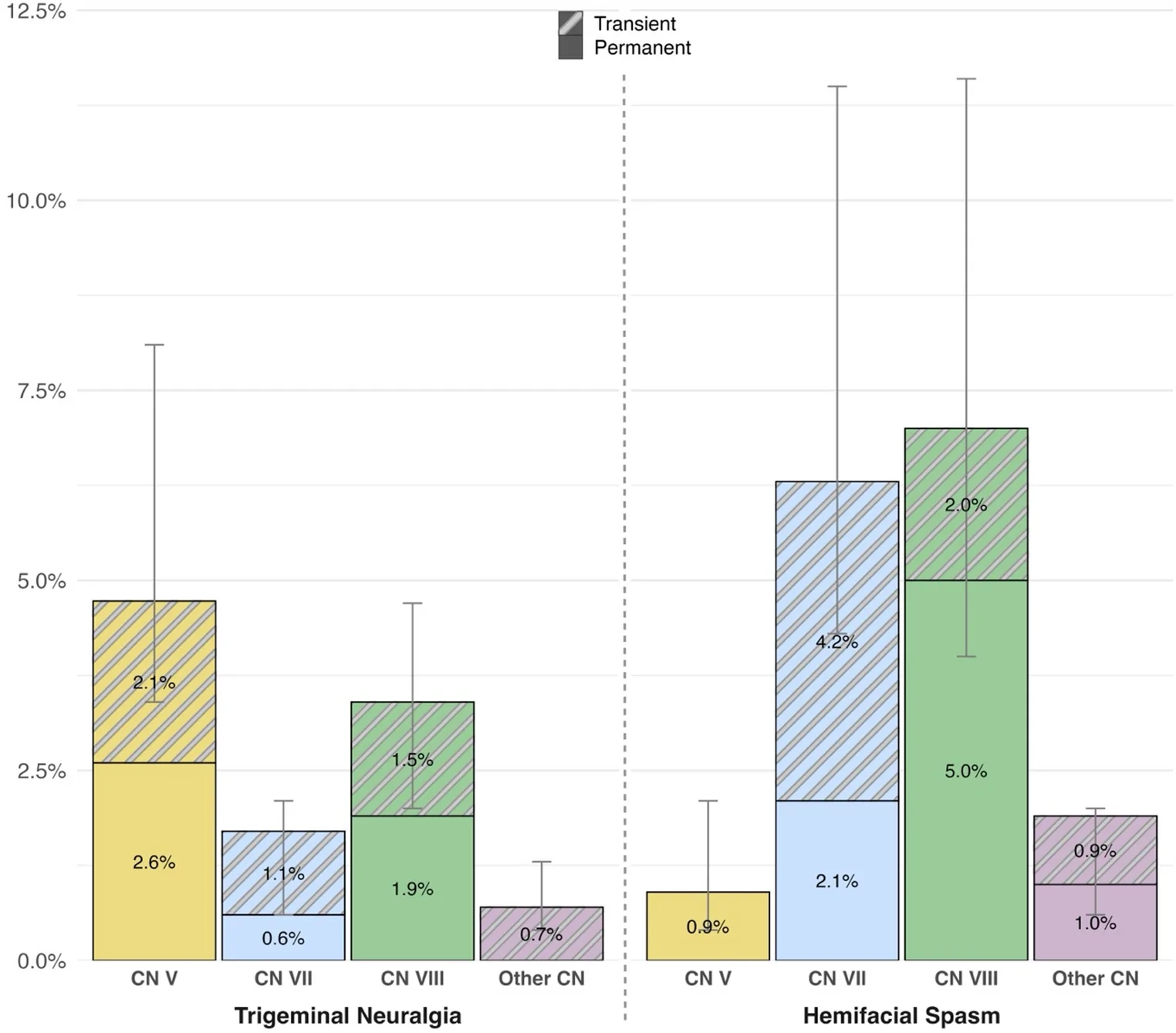
Grouped bar chart illustrating the breakdown of cranial nerve deficits. The two bar clusters correspond to the respective NVCS (TN and HFS). Each bar represents the total rate of a specific CN deficit, with whiskers indicating the 95% CI. The lower solid segment denotes permanent deficits, whereas the upper diagonally patterned segment denotes transient deficits. The “Other CN” category comprises CN IX through XII. Data for GPN was excluded, since only one of the two included studies had reported CN complications. Generated using R

Across 32 studies (5,767 patients), the overall complication rate was 13.9% (95% CI, 0.107–0.180). Permanent neurological deficits occurred in 4.7% of cases (95% CI, 0.030–0.074), all affecting CN. Forest plots of NVCS-specific complication rates are shown in Fig. 4, with detailed breakdowns of complication categories and CN deficits presented in Figs. 5 and 6, respectively. In patients with TN, the most frequent permanent neurological deficit was a sensory CN V deficit, occurring in 2.6% (95% CI, 0.013–0.050), followed by a CN VIII deficit in 1.9% (95% CI, 0.011–0.035). Among patients with HFS, the most frequent permanent deficits were CN VIII in 5.0% (95% CI, 0.026–0.094) and CN VII in 2.1% (95% CI, 0.008–0.050). Subgroup analyses revealed no significant differences in overall complication or permanent neurological deficit rates across NVCS (*p* = 0.23 and *p* = 0.42, respectively). Similarly, no significant differences were observed by complication category. No procedure-related deaths were reported, and none of the studies described cases requiring conversion from a keyhole approach to a standard craniotomy due to limited visibility or maneuverability. Individual forest plots for all remaining primary outcomes are provided in Supplementary Figs. 1–4.

## Discussion

To the best of our knowledge, this is the first systematic review and meta-analysis of MVD surgery with a specific focus on the retrosigmoid keyhole approach. Previous reviews of endoscopic MVD included studies employing smaller craniotomies, but their emphasis was on the visualization technique rather than the surgical approach itself [63]. We conducted a single-arm meta-analysis rather than a classical two-arm comparative analysis, as comparative studies with the standard retrosigmoid craniotomy are lacking in the current literature. Of the 9 included comparative cohort studies, none directly compared keyhole and standard approaches; instead, they addressed other variables such as visualization technique or different patient subgroups. A single-arm meta-analysis allows for systematic pooling of outcomes from uncontrolled studies, helps establish benchmarks, and provides important preliminary evidence in a field where randomized controlled trials or comparative studies are rare or absent. However, to enable comparison of our data with the existing literature on the classic retrosigmoid approach for MVD, we compiled the most relevant studies in a comparative table (Table 2).

**Table 2:** Comparison of findings from this review with selected studies using a standard craniotomy. The most relevant literature is summarized, presenting efficacy and safety outcomes from studies using a standard craniotomy. Corresponding results from the current meta-analysis are shown alongside these data to allow for direct comparison [3, 4, 22, 34, 63]

Authors	Approach	Year	Study type	NVCS	Number of patients	Complete resolution	All complications	Transient CN deficit	Permanent CN deficit	Infection/wound problem/CSF leak	Hemorrhagic or ischemic complication
**Current review**	Keyhole	2025	Single-arm meta-analysis	TN	2219	89.8%	11.5%	3.5%	3.4%	3.6%	0.9%
Barker et al.	Standard	1996	Observ^e^	TN	1204	75%	23.3%	1.6%	2.3%	2.1%	0.3%
Zagzoog et al.^f^	Standard	2019	Meta-Analysis	TN	5783	81%^g^	19%	13.0%^h^		5%	–
**Current review**	Keyhole	2025	Single-arm meta-analysis	HFS	3625	91.1%	16.7%	6.9%	5.9%	3.2%	0.8%
Barker et al.	Standard	1995	Observ	HFS	648	86%	15.6%	3.9%	6.6%	4.6%	0.5%
Hyun et al.	Standard	2010	Observ	HFS	1174	94.1%	12.3%	10.0%	1.9%	0.3%	0.2%
Miller and Miller^i^	Standard	2012	Systematic Review	HFS	4585	91.7%	–	–	–	–	–

^e^ Observ: Observational

^f^ Only “Open MVD” arm

^g^ Complete and very good resolution

^h^ Transient *and* permanent CN deficits

^i^ Yuan et al. deducted from results as it is part of this review itself

The reported complete symptom resolution rates for TN and HFS following keyhole MVD exceed those reported in two large observational studies by Barker et al. from the 1990s, which found rates of 75% for TN and 86% for HFS after standard retrosigmoid craniotomy at one-year FU (Table 2) [3, 4]. When compared with more recent literature, the complete symptom resolution rates after keyhole MVD fall within the expected range. For example, Zagzoog et al. reported a complete resolution rate of 81% in TN patients undergoing MVD via a standard retrosigmoid craniotomy [63]. In HFS, complete resolution rates after MVD through a standard retrosigmoid craniotomy range between 86% and 94% (Table 2) [22, 34]. 

The overall complication rate of keyhole MVD is comparable to that reported in previous studies using the standard retrosigmoid approach (Table 2). Similarly, the rate of permanent CN deficits aligns with the existing literature. For instance, Barker et al. reported rates of 2.3% for TN and 6.6% for HFS, whereas Hyun et al. reported a rate of only 1.9% for their HFS patients [3, 4, 22]. The permanent CN deficit rate observed in our meta-analysis is likely overestimated due to inconsistent reporting on whether CN deficits were transient or permanent. Interestingly, Zagzoog et al. reported CN VII and VIII deficit rates of 9.0% and 4.0%, respectively, among patients with TN treated via a conventional retrosigmoid craniotomy, which were higher than those observed in the TN cohort of the present keyhole analysis (1.7% and 3.4%, respectively) [63]. This difference may reflect a potential advantage of the keyhole approach: by minimizing the exposure of CN VII and VIII, the surgical trajectory is more perpendicular to CN V, potentially reducing unintentional mechanical trauma to CN VII and VIII during MVD for TN.

Rates for hemorrhagic and ischemic complications, as well as for infections, wound problems, and cerebrospinal fluid (CSF) leakages, all remain within the ranges reported in studies using a standard retrosigmoid approach (Table 2). Nonetheless, specific complication rates should be interpreted with caution: while the overall complication rate reflects the proportion of patients experiencing any adverse event, individual complication categories were – in some studies – reported as raw event counts, which may overestimate their actual incidence. Moreover, definitions of complications vary. For instance, some studies count vertigo, tinnitus, and hearing loss separately, whereas others report any involvement of CN VIII as a single complication [55, 64]. 

Recent data from Tomasello et al. further support our findings, reporting a 92.9% complete symptom resolution rate at 1 year and a 5.8% overall complication rate using a 16-mm craniectomy in patients with TN [53]. 

In summary, this meta-analysis shows that keyhole MVD achieves high efficacy, with complete resolution rates comparable to or exceeding those of the standard craniotomy approach. Outcomes were favorable across all NVCS types, and complication rates were similar to existing reports, indicating no added risk from the keyhole technique. None of the studies described cases requiring conversion from a keyhole approach to a standard craniotomy due to limited visibility or maneuverability. Although this study was not designed to demonstrate non-inferiority of the keyhole technique, the results of this analysis suggest that such an assumption might be reasonable.

### Limitations and future directions

Included studies were predominantly of retrospective nature. The mean MINORS score was 9.9, indicating an overall moderate methodological quality among the included studies. Hence, the results of the review should be interpreted with regard to the limitations of the included studies. Additionally, in our review, the number of HFS patients was 1.5 times that of TN patients, although epidemiologically TN is reported to be at least four times more common than HFS. This bias was introduced by a few large studies that included only patients with HFS [35, 55, 61]. 

There was substantial heterogeneity amongst the main outcomes reported in the included studies (with I^2^ values of > 75% for both the overall complication rate and the complete resolution rate) [20]. This suggests that the results of the included studies vary significantly. Thus, the pooled estimates must be interpreted with caution. However, Cochran’s Q test showed no statistically significant differences between the different NVCS subtypes. Furthermore, the observed heterogeneity confirms our choice of the random-effects model.

Heterogeneity may be attributed to several factors. For instance, visualization technique varied across studies, with studies using the microscope, endoscope and/or exoscope for their surgeries. Differences in experience and number of operating surgeons per study might also explain between-study variance. Some studies only included patients from one experienced surgeon, whereas others reported on the first experiences with the endoscope [21, 26]. Lastly, diagnostic heterogeneity was evident when reading the methodology of the different studies. Certain studies incorporated audiometric evaluations to assess for CN VIII deficit, while others relied more on subjective clinical assessments [35]. This might explain variation in complication rates. All these factors, amongst others, may influence operative outcomes. Solely English full texts were included, which carries a risk of omitting data. Finally, a general publication bias cannot be excluded, as studies with negative or non-significant results might remain unpublished.

Although not directly examined in this review, previous studies have reported several advantages of keyhole approaches in neurosurgery, including reductions in postoperative headache and incisional pain, operative times, and hospital stays, along with better cosmetic outcomes [32, 36, 43, 48, 56]. Future research should prioritize randomized studies comparing the retrosigmoid keyhole approach with the standard craniotomy to validate these benefits for MVD surgery. Such investigations will be important for advancing neurosurgical practice beyond techniques developed in the last century. Continued refinement of minimally invasive techniques is essential for further reducing the surgical footprint and improving patient outcomes.

## Conclusion

This systematic review and single-arm meta-analysis suggests that MVD performed via a keyhole approach is a safe and effective treatment for patients with NVCS. Nevertheless, the findings should be interpreted with caution given the substantial heterogeneity among the included studies. Randomized controlled trials are needed to assess the potential advantages of the keyhole approach over the standard retrosigmoid craniotomy. Such studies will be essential to determine whether the keyhole approach can be established as a standard of care in MVD surgery.

## Supplementary Information

Below is the link to the electronic supplementary material.

Supplementary Material 1 (DOCX 704 KB)

## Data Availability

All data supporting the findings of this study are available within the paper and its Supplementary Information.

## Declarations

### Previous presentations

Part of this work was presented as an electronic poster at the 6th Swiss Federation of Clinical Neuro-Societies meeting on October 29, 2025, in Lausanne, Switzerland

### Clinical trial registration

Not applicable. This study is a systematic review and single-arm meta-analysis and does not involve the conduct of a clinical trial.
