# Supplementary material for: Keyhole retrosigmoid approach for microvascular decompression surgery: Systematic review and single-arm meta-analysis
Source: Neurosurg Rev. 2026 Feb 17;49(1):231. doi: 10.1007/s10143-026-04178-0 (PMC13649873; doi:10.1007/s10143-026-04178-0)
Supplement: Supplementary file 1 [file 10143_2026_4178_MOESM1_ESM.docx]

## **Supplementary Information**

**Article Title**

Keyhole Retrosigmoid Approach for Microvascular Decompression Surgery: Systematic Review and Single-Arm Meta-Analysis

**Journal Name**

Neurosurgical Review

**Authors**

Valentin F. Weiger, MD^1^ (ORCID-ID: 0009-0002-2799-6875); Florian S. Halbeisen, PhD^2^ (ORCID-ID: 0000-0003-2755-9501); Felipe Constanzo, MD^3^ (ORCID-ID: 0000-0002-6736-7195); Michel Roethlisberger, MD^1^ (ORCID-ID: 0000-0003-0932-0193); Jehuda Soleman, MD^1^ (ORCID-ID: 0000-0003-1900-9286); Luigi Mariani, MD^1^ (ORCID-ID: 0000-0002-1971-5095); Raphael Guzman, MD^1^ (ORCID-ID: 0000-0001-9620-0581); Jonathan Rychen, MD^1^ (ORCID-ID: 0000-0002-1121-6996)

^1^Department of Neurosurgery, University Hospital of Basel, Basel, Switzerland

^2^Surgical Outcome Research Center Basel, University Hospital of Basel, Basel, Switzerland

^3^Department of Skull Base Surgery, Clinica Bio Bio, Concepcion, Chile

### Supplementary Data

**MeSH Terms**

*Microvascular Decompression Surgery* (MeSH ID: D061145, Source: National Library of Medicine, 2025)

**Search strings**

1) For PubMed:

((Trigeminal neuralgia OR Hemifacial spasm OR Glossopharyngeal neuralgia OR Vestibular paroxysmia OR Nervus intermedius neuralgia OR neurovascular disorder) AND

(Microvascular decompression surgery OR vascular decompression OR microvascular decompression))

OR

((retrosigmoid* OR retromastoid*) AND (keyhole OR burr* OR minimally invasive))

2) For Embase via query translator (includes MEDLINE):

(('trigeminal neuralgia' OR 'hemifacial spasm' OR 'glossopharyngeal neuralgia' OR 'vestibular paroxysmia' OR 'nervus intermedius neuralgia' OR 'neurovascular disorder') AND

('microvascular decompression surgery' OR 'vascular decompression' OR 'microvascular decompression'))

OR

(('retrosigmoid*' OR 'retromastoid*') AND ('keyhole' OR 'burr*' OR 'minimally invasive'))

### Supplementary Figures

**Supplementary figure 1: Forest plot of partial resolution rates**

Each included study is shown with the first author’s name, the number of patients with partial resolution of symptoms (events), and total number of patients enrolled. The plot depicts the point estimate of the partial resolution rate for each study, together with its 95% CI and study weight in the meta-analysis. Studies are grouped according to NVCS types. Overall pooled results of the random-effects meta-analysis, along with heterogeneity and potential subgroup differences are presented.

**Supplementary figure 2: Forest plot of failure rates**

Each included study is shown with the first author’s name, the number of patients with failure outcome (events), and total number of patients enrolled. The plot depicts the point estimate of the failure rate for each study, together with its 95% CI and study weight in the meta-analysis. Studies are grouped according to NVCS types. Overall pooled results of the random-effects meta-analysis, along with heterogeneity and potential subgroup differences are presented.

**Supplementary figure 3: Forest plot of recurrence rates**

Each included study is shown with the first author’s name, the number of patients with recurrence of symptoms (events), and total number of patients enrolled. The plot depicts the point estimate of the recurrence rate for each study, together with its 95% CI and study weight in the meta-analysis. Studies are grouped according to NVCS types. Overall pooled results of the random-effects meta-analysis, along with heterogeneity and potential subgroup differences are presented.

**Supplementary figure 4: Forest plot of permanent cranial nerve deficit rates**

Each included study is shown with the first author’s name, the number of patients with permanent cranial nerve (CN) deficit (events), and total number of patients enrolled. The plot depicts the point estimate of the permanent cranial nerve deficit rate for each study, together with its 95% CI and study weight in the meta-analysis. Studies are grouped according to NVCS types. Overall pooled results of the random-effects meta-analysis, along with heterogeneity and potential subgroup differences are presented.

**Supplementary figure 1: Forest plot of partial resolution rates**

**Supplementary figure 2: Forest plot of failure rates**

**Supplementary figure 3: Forest plot of recurrence rates**

**Supplementary figure 4: Forest plot of permanent cranial nerve (CN) deficit rates**

### Supplementary Tables

Supplementary table 1: **Qualitative analysis**

| **Authors, year** | **Country** | **Type of Study** | | **MINORS score** | **Selection bias** | **Confounding** | **Performance and detection bias** | **Attrition bias** | **Reporting bias** | **No. of patients** | **No. of patients lost to FU** |
| --- | --- | --- | --- | --- | --- | --- | --- | --- | --- | --- | --- |
| **Chen et al.**, 2008 | China | Observational | Retro | 10 | High | High | High | Low | Low | 156 | 0 |
| **El-Garem et al.**, 2002 | France | Observational | Retro | 10 | High | High | High | Low | Low | 42 | 0 |
| **Ferroli et al.**, 2010 | Italy | Comparative 2-arm | Retro | 8 | High | High | Low | High | Low | 476 | 75 |
| **Fu et al.**, 2024 | China | Comparative 2-arm | Retro | 9 | Low | High | Low | Low | Low | 168 | 12 |
| **Guo et al.**, 2024 | China | Observational | Retro | 10 | High | High | High | Low | Low | 189 | 0 |
| **Jarrahy et al.**, 2000 | United States | Observational | Retro | 10 | High | High | High | Low | Low | 21 | 0 |
| **Pak et al.**, 2022 | UK | Observational | Retro | 11 | Low | High | High | Low | Low | 25 | 0 |
| **Rossini et al.**, 2024 | Italy | Observational | Retro | 10 | High | High | High | Low | Low | 7 | 0 |
| **Sun et al.**, 2020 | China | Observational | Retro | 10 | Low | High | High | Low | Low | 20 | 0 |
| **Teo et al.**, 2006 | Australia | Observational | Retro | 10 | High | High | High | Low | Low | 114 | 0 |
| **Yang and Wang**, 2019 | China | Comparative 2-arm | Retro | 8 | Low | High | High | Low | Low | 298 | 15 |
| **Yang et al.**, 2014 | China | Comparative 2-arm | Retro | 11 | Low | High | High | Low | Low | 223 | 5 |
| **Yu et al.**, 2024 | China | Comparative 2-arm | Retro | 10 | High | High | High | Low | Low | 297 | 0 |
| **Artz et al.**, 2008 | United States | Observational | Retro | 9 | High | High | High | Low | Low | 19 | 0 |
| **Ma et al.**, 2010 | China | Observational | Retro | 12 | Low | High | Low | Low | Low | 200 | 0 |
| **Cheng et al.**, 2008 | Taiwan | Observational | Retro | 11 | High | High | High | Low | Low | 32 | 0 |
| **Eby et al.**, 2001 | United States | Observational | Retro | 7 | High | High | High | Low | Low | 3 | 0 |
| **Guo et al.**, 2021 | China | Observational | Retro | 9 | High | High | High | Low | Low | 81 | 0 |
| **Huang et al.**, 2021 | China | Observational | Retro | 10 | High | High | High | Low | Low | 148 | 0 |
| **Iwami et al.**, 2021 | Japan | Observational | Retro | 11 | Low | High | High | Low | Low | 20 | 0 |
| **Li et al.**, 2018 | China | Observational | Retro | 10 | Low | High | High | Low | Low | 320 | 0 |
| **Liu et al.**, 2016 | China | Observational | Retro | 11 | High | High | High | Low | Low | 12 | 0 |
| **Magnan et al.**, 1997 | France | Observational | Retro | 7 | High | High | High | High | Low | 59 | 6 |
| **Montava et al.**, 2016 | France | Observational | Retro | 10 | High | High | High | Low | Low | 434 | 0 |
| **Ricci et al.**, 2019 | Italy | Observational | Prosp | 11 | High | High | High | Low | Low | 12 | 0 |
| **Wang et al. (D. Wang)**, 2021 | China | Comparative 2-arm | Prosp | 12 | Low | Low | High | Low | Low | 984 | 3 |
| **Sun et al.**, 2021 | China | Comparative 2-arm | Retro | 10 | High | High | High | Low | Low | 205 | 0 |
| **Yuan et al.**, 2005 | China | Observational | Retro | 10 | High | High | High | Low | Low | 1200 | 0 |
| **Yun et al.**, 2024 | South Korea | Observational | Retro | 11 | High | High | High | Low | Low | 25 | 0 |
| **Zhu et al.**, 2021 | China | Comparative 1-arm | Retro | 12 | High | High | Low | Low | Low | 54 | 0 |
| **Blue et al.**, 2020 | United States | Observational | Retro | 7 | High | High | High | Low | Low | 2 | 0 |
| **Wang et al. (J. Wang)**, 2018 | China | Comparative 2-arm | Retro | 10 | High | High | High | Low | Low | 37 | 0 |

**Abbreviations:**

No.: Number

FU: Follow-up

Retro: Retrospective

Prosp: Prospective

Supplementary table 2: **Quantitative analysis**

| **Authors, year** | **Exclusion of subgroup** | **No. of patients** | **M** | **F** | **Mean age [yrs]** | **Lower limit** | **Upper limit** | **No. of TN** | **No. of HFS** | **No. of GPN** | **Type of procedure** | **Visualization technique** | **Mean FU [months]** | **No. of patients lost to FU** | **Complete Resolution** | **Partial Resolution** | **Failure** | **Recurrence** | **No. of patients with complications** | **Permanent CN deficit** | **Transient CN deficit** | **Infection / Wound problem / CSF leak** | **Hemorrhagic or ischemic complication** | **Other complications** |
| --- | --- | --- | --- | --- | --- | --- | --- | --- | --- | --- | --- | --- | --- | --- | --- | --- | --- | --- | --- | --- | --- | --- | --- | --- |
| **Chen et al.**, 2008 | 11 patients excluded | 156 | n/a | n/a | 57.2 | 26 | 85 | 156 | 0 | 0 | MVD | EA | 18.0 | 0 | 140 | 5 | 11 | 0 | 9 | 0 | 0 | 5 | 4 | 0 |
| **El-Garem et al.**, 2002 |  | 42 | 20 | 22 | 58.2 | n/a | n/a | 42 | 0 | 0 | MVD | EA | n/a | 0 | 31 | 4 | 3 | 4 | 2 | 0 | 0 | 1 | 0 | 1 |
| **Ferroli et al.**, 2010 |  | 476 | 214 | 262 | 60.0 | 19 | 82 | 476 | 0 | 0 | MVD | Micro | 70.5 | 75 | 335 | 11 | n/a | n/a | 67 | 47 | 0 | 19 | 1 | 0 |
| **Fu et al.**, 2024 | HFS patients excluded | 168 | 60 | 108 | 53.7 | n/a | n/a | 168 | 0 | 0 | MVD | Endo | n/a | 12 | 136 | n/a | n/a | 5 | 26 | 17 | 9 | 0 | 0 | 0 |
| **Guo et al.**, 2024 |  | 189 | 90 | 99 | 58.0 | 29 | 75 | 189 | 0 | 0 | MVD | Endo | 27.0 | 0 | 178 | 5 | 2 | 4 | 21 | 2 | 17 | 2 | 0 | 0 |
| **Jarrahy et al.**, 2000 |  | 21 | 8 | 13 | 57.0 | 32 | 86 | 21 | 0 | 0 | MVD | EA | 6.0 | 0 | 15 | 3 | 3 | 0 | 5 | 0 | 0 | 6 | 0 | 0 |
| **Pak et al.**, 2022 |  | 25 | 10 | 15 | 63.0 | n/a | n/a | 25 | 0 | 0 | MVD | Endo | n/a | 0 | 25 | 0 | 0 | 3 | 2 | 2 | 2 | 0 | 0 | 0 |
| **Rossini et al.**, 2024 | 2 patients excluded | 7 | 3 | 4 | 64.4 | 43 | 79 | 7 | 0 | 0 | MVD | Exoscopic | 4.4 | 0 | 7 | 0 | 0 | 0 | 1 | 0 | 0 | 1 | 0 | 0 |
| **Sun et al.**, 2020 |  | 20 | 8 | 12 | 55.0 | 48 | 71 | 20 | 0 | 0 | MVD | Endo | 18.0 | 0 | 16 | 4 | 0 | 0 | 2 | 0 | 2 | 0 | 0 | 0 |
| **Teo et al.**, 2006 |  | 114 | 66 | 48 | 54.0 | 34 | 76 | 114 | 0 | 0 | MVD | EA + Micro | 38.0 | 0 | 113 | 1 | 0 | 0 | 10 | 1 | 8 | 1 | 0 | 0 |
| **Yang and Wang**, 2019 |  | 298 | 142 | 156 | n/a | 36 | 81 | 298 | 0 | 0 | MVD, IN | Micro | n/a | 15 | 251 | 21 | 11 | 11 | 43 | 0 | 9 | 8 | 0 | 0 |
| **Yang et al.**, 2014 |  | 223 | 106 | 117 | 59.5 | 36 | 89 | 223 | 0 | 0 | MVD | Micro | 44.2 | 5 | 206 | 12 | 0 | 20 | 15 | 9 | 0 | 4 | 0 | 2 |
| **Yu et al.**, 2024 |  | 297 | 119 | 187 | 60.7 |  |  | 297 | 0 | 0 | MVD | Micro, Endo | 48.9 | 0 | n/a | n/a | n/a | n/a | 47 | 36 | 0 | 11 | 0 | 0 |
| **Artz et al.**, 2008 | 1 patient excluded | 19 | n/a | n/a | n/a |  |  | 14 | 5 | 0 | MVD | Endo | 14.0 | 0 | 12  5 | 2  0 | 0  0 | 0  1 | 3 | 1 | 1 | 1 | 0 | 0 |
| **Ma et al.**, 2010 | GPN patients excluded | 200 | 78 | 122 | 62.5 |  |  | 169 | 31 | 0 | MVD, IN | Micro | n/a | 0 | 158  26 | n/a  n/a | n/a  n/a | 6  4 | 61 | 3 | 38 | 10 | 1 | 0 |
| **Cheng et al.**, 2008 |  | 32 | 12 | 20 | 55.0 | 30 | 80 | 0 | 32 | 0 | MVD | EA + Endo | 57.6 | 0 | 31 | 1 | 0 | 0 | 2 | 1 | 1 | 0 | 0 | 0 |
| **Eby et al.**, 2001 |  | 3 | 1 | 2 | 64.3 | 51 | 72 | 0 | 3 | 0 | MVD | Endo | 8.3 | 0 | 3 | 0 | 0 | 0 | 2 | 0 | 1 | 0 | 0 | 1 |
| **Guo et al.**, 2021 |  | 81 | 22 | 59 | 52.0 | 20 | 76 | 0 | 81 | 0 | MVD | Endo | n/a | 0 | 77 | 0 | 4 | 0 | 8 | 4 | 4 | 0 | 0 | 0 |
| **Huang et al.**, 2021 |  | 148 | 58 | 90 | n/a | 21 | 71 | 0 | 148 | 0 | MVD | Micro | n/a | 0 | 143 | n/a | n/a | 1 | 23 | 14 | 6 | 2 | 1 | 0 |
| **Iwami et al.**, 2021 |  | 20 | 7 | 13 | 56.2 |  |  | 0 | 20 | 0 | MVD | Endo | 11.7 | 0 | 19 | 1 | 0 | 0 | 1 | 0 | 1 | 0 | 0 | 0 |
| **Li et al.**, 2018 |  | 320 | 95 | 225 | 49.3 | 23 | 73 | 0 | 320 | 0 | MVD | Micro | n/a | 0 | 286 | 23 | 11 | 0 | 6 | 0 | 3 | 3 | 0 | 0 |
| **Liu et al.**, 2016 |  | 12 | 4 | 8 | 51.0 | 34 | 62 | 0 | 12 | 0 | MVD | Micro | 54.0 | 0 | 12 | 0 | 0 | 0 | 3 | 3 | 3 | 0 | 0 | 0 |
| **Magnan et al.**, 1997 | 1 patient excluded | 59 | n/a | n/a | n/a | n/a | n/a | 0 | 59 | 0 | MVD | EA | 14.0 | 6 | 40 | n/a | n/a | 0 | 9 | 3 | 5 | 4 | 0 | 0 |
| **Montava et al.**, 2016 | Only first surgery patients included | 434 | n/a | n/a | n/a | n/a | n/a | 0 | 434 | 0 | MVD | EA | n/a | 0 | 337 | 20 | 59 | 18 | 83 | 16 | 37 | 40 | 3 | 0 |
| **Ricci et al.**, 2019 |  | 12 | 5 | 7 | 57.5 | 49 | 71 | 0 | 12 | 0 | MVD | EA | n/a | 0 | 12 | 0 | 0 | 1 | 2 | 0 | 0 | 2 | 0 | 0 |
| **Wang et al. (D. Wang)**, 2021 |  | 984 | 355 | 629 | 53.2 | n/a | n/a | 0 | 984 | 0 | MVD | Micro | n/a | 3 | 899 | 55 | 27 | 0 | 50 | 18 | 18 | 10 | 4 | 0 |
| **Sun et al.**, 2021 |  | 205 | 63 | 142 | n/a | 22 | 73 | 0 | 205 | 0 | MVD | Micro | n/a | 0 | 199 | 5 | 1 | 13 | 26 | 2 | 23 | 0 | 1 | 0 |
| **Yuan et al.**, 2005 |  | 1200 | 485 | 715 | 53.0 | 23 | 72 | 0 | 1200 | 0 | MVD | Micro | 50.4 | 0 | 1064 | 67 | 31 | 38 | 222 | 168 | 0 | 35 | 0 | 19 |
| **Yun et al.**, 2024 |  | 25 | 9 | 16 | n/a | 35 | 72 | 0 | 25 | 0 | MVD | Endo | n/a | 0 | 25 | 0 | 0 | 2 | 10 | 1 | 8 | 1 | 0 | 0 |
| **Zhu et al.**, 2021 | M-Group excluded | 54 | 8 | 46 | 51.5 | n/a | n/a | 0 | 54 | 0 | MVD | Endo | n/a | 0 | 44 | 4 | 6 | 0 | 26 | 26 | 4 | 2 | 0 | 0 |
| **Blue et al.**, 2020 |  | 2 | 1 | 1 | 57.0 | 53 | 61 | 0 | 0 | 2 | MVD | Endo | 13.5 | 0 | 2 | 0 | 0 | 0 | 0 | 0 | 0 | 0 | 0 | 0 |
| **Wang et al. (J. Wang)**, 2018 |  | 37 | 18 | 19 | n/a | 40 | 81 | 0 | 0 | 37 | MVD, MVD + RT | Micro | n/a | 0 | 33 | 4 | 0 | 0 | 7 | 2 | 2 | 3 | 0 | 0 |

**Abbreviations:**

No.: Number

FU: Follow-up

MVD: Microvascular decompression

IN: Internal neurolysis

PR: Partial rhizotomy

Micro: Microscopic

Endo: Endoscopic

EA: Endoscopic assisted
